# Chemogenomic Screen for Imipenem Resistance in Gram-Negative Bacteria

**DOI:** 10.1128/mSystems.00465-19

**Published:** 2019-11-19

**Authors:** Jessica Y. El Khoury, Alexandra Maure, Hélène Gingras, Philippe Leprohon, Marc Ouellette

**Affiliations:** a Axe des Maladies Infectieuses et Immunitaires du Centre de Recherche du CHU de Québec and Département de Microbiologie, Infectiologie et Immunologie, Faculté de Médecine, Université Laval, Québec, Québec, Canada; University of Illinois at Chicago

**Keywords:** chemical mutagenesis, carbapenem resistance, *Escherichia coli*, *Klebsiella pneumoniae*, *Pseudomonas aeruginosa*

## Abstract

Carbapenem-resistant Gram-negative bacteria are considered a major threat to global health. Imipenem (IMP) is used as a last line of treatment against these pathogens, but its efficacy is diminished by the emergence of resistance. We applied a whole-genome screen in Escherichia coli, Klebsiella pneumoniae, and Pseudomonas aeruginosa isolates that were submitted to chemical mutagenesis, selected for IMP resistance, and characterized by next-generation sequencing. A comparative analysis of IMP-resistant clones showed that most of the highly mutated genes shared by the three species encoded proteins involved in transcription or signal transduction. Of these, the *rpoD* gene was one of the most prevalent and an E. coli strain disrupted for *rpoD* displayed a 4-fold increase in resistance to IMP. E. coli and K. pneumoniae also specifically shared several mutated genes, most involved in membrane/cell envelope biogenesis, and the contribution in IMP susceptibility was experimentally proven for amidases, transferases, and transglycosidases. P. aeruginosa differed from the two *Enterobacteriaceae* isolates with two different resistance mechanisms, with one involving mutations in the *oprD* porin or, alternatively, in two-component systems. Our chemogenomic screen performed with the three species has highlighted shared and species-specific responses to IMP.

**IMPORTANCE** Gram-negative carbapenem-resistant bacteria are a major threat to global health. The use of genome-wide screening approaches to probe for genes or mutations enabling resistance can lead to identification of molecular markers for diagnostics applications. We describe an approach called Mut-Seq that couples chemical mutagenesis and next-generation sequencing for studying resistance to imipenem in the Gram-negative bacteria Escherichia coli, Klebsiella pneumoniae, and Pseudomonas aeruginosa. The use of this approach highlighted shared and species-specific responses, and the role in resistance of a number of genes involved in membrane biogenesis, transcription, and signal transduction was functionally validated. Interestingly, some of the genes identified were previously considered promising therapeutic targets. Our genome-wide screen has the potential to be extended outside drug resistance studies and expanded to other organisms.

## Abstract (precis)

Gram-negative carbapenem-resistant bacteria are a major threat to global health. The use of genome-wide screening approaches to probe for genes or mutations enabling resistance can lead to identification of molecular markers for diagnostics applications. We describe an approach called Mut-Seq that couples chemical mutagenesis and next-generation sequencing for studying resistance to imipenem in the Gram-negative bacteria Escherichia coli, Klebsiella pneumoniae, and Pseudomonas aeruginosa. The use of this approach highlighted shared and species-specific responses, and the role in resistance of a number of genes involved in membrane biogenesis, transcription, and signal transduction was functionally validated. Interestingly, some of the genes identified were previously considered promising therapeutic targets. Our genome-wide screen has the potential to be extended outside drug resistance studies and expanded to other organisms.

## INTRODUCTION

The World Health Organization (WHO) refers to antimicrobial resistance (AMR) as a major threat to global health and has established a list of priority pathogens for research and development of new effective antibiotics (1). Among the urgent threats is carbapenem resistance in the Gram-negative species Escherichia coli and Klebsiella pneumoniae. Both are commensal bacteria of the gastrointestinal tract of human and animals; they harbor many virulence factors and are responsible for different types of infections (2, 3). Another threat is represented by the opportunistic pathogen Pseudomonas aeruginosa (4). Those three bacterial species are leading causes of hospital-acquired infections (5).

Carbapenems such as imipenem (IMP) and meropenem (MEM) are β-lactam antibiotics (6) that bind and inhibit multiple penicillin binding proteins (PBPs) while resisting hydrolysis by class A extended-spectrum β-lactamases and class C β-lactamases (AmpC) (7). Carbapenems are used as a last line of treatment against multidrug-resistant Gram-negative pathogens (7). Nonetheless, resistance against carbapenems has been detected in *Enterobacteriaceae* mainly due to the production of more-potent β-lactamases such as K. pneumoniae carbapenemases (KPCs), class B metallo-β-lactamases (e.g., VIM, NDM, and IMP) and class D (OXA-type) β-lactamases found both on plasmids and in the chromosome (2, 8). Resistance resulting from the loss or modification of porins (OmpK35/36 for K. pneumoniae and OmpC/F for E. coli), in combination with the production of plasmid-encoded or chromosomally encoded AmpC, was also observed (9–12). The loss of the porin OprD in P. aeruginosa constitutes the major driver of carbapenem resistance, although resistance due to class B metallo-β-lactamases was also reported (13–16). β-lactam and β-lactamase inhibitor combinations (e.g., IMP-relebactam, MEM-vaborbactam) are showing promising results *in vitro* and in clinical trials against carbapenem-resistant Gram-negative bacteria (7, 17, 18). Both IMP and MEM target PBPs, and usually high-level resistance to IMP correlates with decreased susceptibility to MEM (19, 20). Studies performed with IMP have been useful for understanding the mode of action and resistance to carbapenems.

Exploring resistance to antibiotics at the genomic level is proving useful at revealing drug targets and modes of action, resistance mechanisms, and genes or mechanisms that play subtler roles such as facilitating resistance or compensating for fitness cost (21–24). The objective of this study was to apply a whole-genome sequencing (WGS) screen for IMP resistance in sensitive isolates of E. coli, K. pneumoniae, and P. aeruginosa. This screen couples chemical mutagenesis, selection for IMP resistance, and the characterization of IMP-resistant clones by next-generation sequencing (NGS). This approach, called “Mut-Seq” (25), has been helpful for various studies, including studies of drug resistance (26, 27). We found that the *rpoD* gene, encoding an RNA polymerase sigma factor, was the most prevalent mutated gene among IMP-resistant clones from the three species, and we experimentally validated its role in IMP resistance in E. coli. Mutations were also detected in several genes related to the cell wall and membrane biogenesis, and these are shown to confer low-level IMP resistance in *Enterobacteriaceae*. Finally, mutations in OprD were frequent in P. aeruginosa but we show that two-component (TC) signal transduction systems are also likely involved in IMP resistance.

## RESULTS

### Chemical mutagenesis and selection for resistance to IMP.

E. coli ATCC 25922, K. pneumoniae ATCC 13883, and P. aeruginosa ATCC 27853 were treated with ethyl methane sulfonate (EMS) and selected for growth in the presence of IMP. The EMS concentrations and exposure and recovery times as well as the IMP concentrations for selection were optimized (see Materials and Methods). The minimum concentration of IMP used for selection was determined as the concentration at which growth occurred in the presence of IMP for the mutagenized populations but not for the nonmutagenized control populations.

The IMP-resistant clones for the three species were between 2-fold and 16-fold more resistant than the respective parental wild-type (WT) clones (Tables 1 and 2; see also Table S1 in the supplemental material). The levels of resistance to IMP measured for most clones of E. coli and K. pneumoniae were considered intermediate (MIC, 2 μg/ml) according to the Clinical and Laboratory Standards Institute (CLSI) guidelines (28). The IMP MIC for two E. coli clones and five K. pneumoniae clones was 4 μg/ml, a level consistent with clinical resistance (Table 1 and Table S1, respectively). According to the MIC breakpoints for P. aeruginosa, all but one clone reached resistance levels consistent with clinical resistance (MIC, ≥8 μg/ml) (Table 2). We observed that cells with higher IMP MICs also showed decreased susceptibilities to MEM (Table 3). The MEM MICs increased by 2-fold, 4-fold, and up to 16-fold in IMP-selected K. pneumoniae, E. coli, and P. aeruginosa, respectively (Table 3). The MEM resistance in P. aeruginosa reached levels consistent with clinical resistance.

**TABLE 1:** IMP MICs for E. coli ATCC 25922 mutants and amino acid substitutions detected in genes mutated in at least three mutants

Strain^*a*^	IMP MIC (μg/ml)^*b*^	Gene ID and mutation^*c*^												
		DR76 3827 (*yceG*)	DR76 2948	DR76 1787 (*amiC*)	DR76 1419 (*rpoD*)	DR76 3839 (*rne*)	DR76 3362	DR76 1882 (*nlpD*)	DR76 689 (*wecA*)	DR76 475	DR76 727 (*gidA*)	DR76 4272 (*tolA*)	DR76 2503 (*slt*)	DR76 839 (*spoT*)
WT	0.25													
M27	0.5								G163D					
M38	0.5		P380L											
M5	1	A212T		G402R										
M6	1		P380L		A72V									
M7	1	Y274H	**P380L**											
M10	1					Q705*								
M16	1													
M17	1							276 Ins						
M18	1													
M19	1					R335C								
M20	1			**G402R**							**A539V**			
M24	1		P380L	A203T								A223T		
M25	1		P380L	E229K							A77T			
M28	1	W162*												
M33	1	S230L								A122V			121 ins	
M39	1		P380L	G197S										
M40	1		P380L											
M42	1													
M44	1						D113N			P101S				
M46	1		P380L											
M49	1		P380L					D321G						
M50	1	P281S	P380L	Q353*										
M1	2	E224K				Q692*								
M2	2	M1I								**A254V**				
M3	2	T273I				Q775*								
M4	2				A444V		D318A		G45R					
M8	2		P380L										W455*	
M9	2							276 Ins						
M11	2	W156*				P250L Q691*						**M67I**		
M12	2				A444T				**R40C**					
M13	2				I457L				D159N					
M15	2	Q92*												
M21	2										R204C			
M22	2		P380L					342 Del						
M23	2		D376N		**A444V**									
M29	2	P246L					**A429T**							
M30	2	T273I												
M31	2	289 Ins												
M32	2	Q92*												P168S
M34	2	P246L					A429T							
M35	2	W24*				Q649*								
M36	2						D113N			P101S				
M37	2	W193*								A149V				
M41	2			Q57*										
M43	2				A444V									
M45	2			R102C	A444V							G316E		
M47	2	Q92*									V102M			
M48	2	E107K					A259V	Q161*						
M14	4	S230L	P380L	Q15*									R475*	**A138V**
M26	4				A444T				G51E					

a Mutants are listed in ascending order of IMP MIC.

b MICs were monitored with at least three biological replicates. For all differences of 2-fold or higher, there was no variability in the observed MICs.

c Mutations correspond to amino acid substitutions, and numbers refer to amino acid positions in the protein. In the case of small insertions (Ins) or deletions (Del), the number indicated refers to the nucleotide position at which these occurred in the gene. Asterisks denote stop codons. ID, identifier. Mutations indicated in bold have been functionally tested for their role in IMP resistance by individual transformation in E. coli ATCC 25922 (see Table 5).

**TABLE 2:** IMP MICs for P. aeruginosa ATCC 27853 mutants and amino acid substitutions detected in genes mutated in two or more mutants

Strain^*a*^	IMP MIC (μg/ml)^*b*^	Gene ID^*c*^				
		A4W92_06800 (*oprD*)	A4W92_13070 (sensor HK)^*d*^	A4W92_13065 (response regulator)	A4W92_04840 (sensor HK)^*d*^	A4W92_05675 (*phoQ*)
WT	2					
M2	4	G183D				
M44	8	S325F				
M13	8	G402D				
M31	8			A174V		
M35	8			A174V		
M40	8			A174V		
M47	8			M53V		
M36	8					E198K
M8	8		A252V			
M39	8		A252V			
M23	8		G260D			
M30	8		G260D			
M43	8		G260D			
M37	8		V268M			
M28	8		S285F			
M25	8		L303F			
M1	8		R419H			
M3	8		R419H			
M11	16		R419H			
M5	16		A252V			Q258*
M12	16		E291K			
M14	16		E291K			
M16	16		E291K			
M6	16				R419H	
M9	16				R419H	
M4	16	W6*				
M10	16	W6*				
M18	16	W6*				
M22	16	W6*				
M33	16	W6*				
M26	16	Q19*				
M32	16	Q19*				
M29	16	Q30*				
M17	16	W65*				
M20	16	Q67*				
M48	16	Q67*				
M38	16	Q79*				
M24	16	W138*				
M27	16	W138*				
M45	16	W138*				
M46	16	W138*				
M7	16	Q158*				
M50	16	Q158*				
M42	16	Q235*				
M21	16	W277*				
M41	16	Q295*				
M19	16	Q296*				
M15	16	W339*				
M34	16	Y343N				
M49	16	W415*				

a Mutants are listed in ascending order of IMP MIC and grouped by mutation profiles.

b MICs were monitored with at least three biological replicates. For all differences of 2-fold or higher, there was no variability in the observed MICs.

c Mutations correspond to amino acid substitutions, and numbers refer to amino acid position in the protein. Asterisks denote stop codons.

d HK, histidine kinase.

**TABLE 3:** IMP and MEM MICs for some selected mutants of E. coli, K. pneumoniae, and P. aeruginosa

Strain	MIC (μg/ml)^*a*^	
	IMP	MEM
E. coli		
ATCC 25922	0.25	0.03
M11	2	0.03
M14	4	0.12
M23	2	0.06
M26	4	0.12

K. pneumoniae		
ATCC 13883	1	0.06
M6	4	0.12
M9	4	0.12
M17	2	0.06
M18	2	0.12
M21	2	0.06
M40	2	0.06

P. aeruginosa		
ATCC 27853	2	0.5
M2	4	2
M4	16	8
M5	16	8
M6	16	4
M31	8	2
M34	16	4
M36	8	4
M37	8	2

a MICs were monitored with at least three biological replicates. For all differences of 2-fold or higher, there was no variability in the observed MICs.

10.1128/mSystems.00465-19.2 TABLE S1 IMP MICs for K. pneumoniae ATCC 13883 mutants and mutations in genes shared with E. coli mutants. Download Table S1, PDF file, 0.08 MB. Copyright © 2019 El Khoury et al. 2019 El Khoury et al. This content is distributed under the terms of the Creative Commons Attribution 4.0 International license.

### Clusters of orthologous groups of mutated proteins.

**FIG 1:**
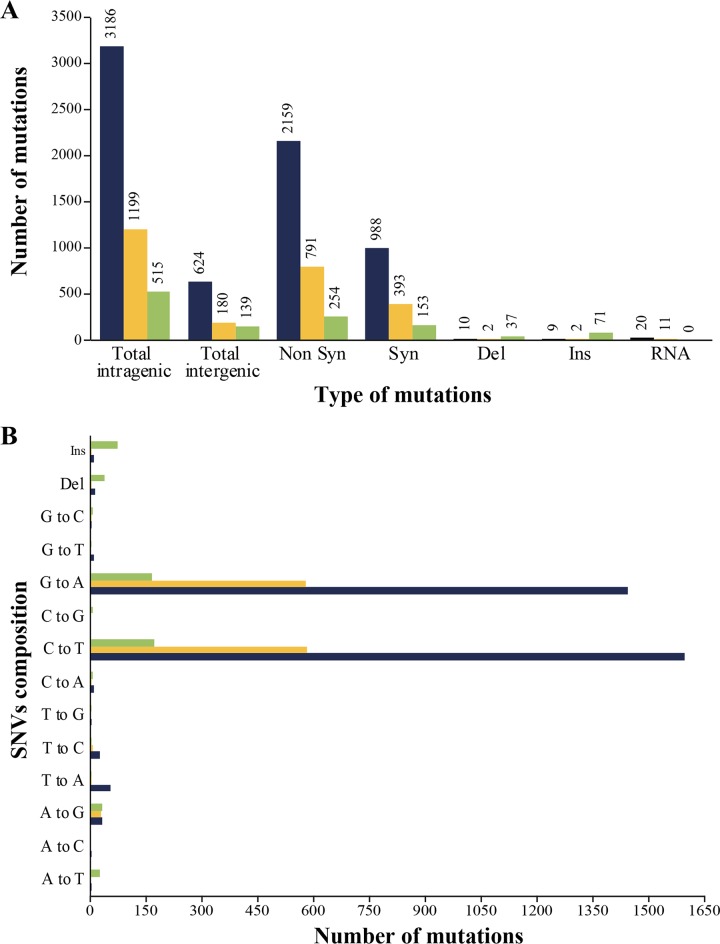
Summary of number and type of mutations induced by EMS in the three bacterial species. (A and B) Number and type (A) as well as distribution (B) of mutations found in Mut-Seq mutants selected for IMP resistance in E. coli (blue), K. pneumoniae (orange), and P. aeruginosa (green). Syn, synonymous; Non Syn, nonsynonymous; Del, deletion; Ins, insertion; RNA, rRNA, tRNA, transfer-messenger RNA (tmRNA), and noncoding RNA (ncRNA) (inclusive); SNV, single nucleotide variant.

The genomes of 145 IMP-resistant clones (45 K. pneumoniae, 50 E. coli, and 50 P. aeruginosa clones) were analyzed by NGS. The genomes of the parent WT strains were also sequenced. A total of 3,810 single nucleotide (nt) variants (SNVs) were identified in the E. coli mutants (Fig. 1A), with a majority of the clones having at least 40 SNVs (Table S2). In K. pneumoniae, 1,379 SNVs were detected (Fig. 1A), with an average of 22 mutations (range, 5 to 39) per genome (Table S3). In P. aeruginosa, 654 SNVs were identified (Fig. 1A), with an average of 10 mutations (range, 2 to 18) found per mutant (Table S4). SNVs were more prevalent in coding regions than in intergenic regions for all species, and nonsynonymous SNVs predominated among coding mutations (Fig. 1A). While we cannot exclude the possibility that some SNVs resulted from IMP selection alone, it is highly likely that most were instead genuinely induced by EMS treatment. First, resistant clones were obtained using experimental conditions optimized such that no clone would grow upon IMP selection if the culture was not initially mutagenized by EMS. Second, most SNVs consisted of G-to-A and C-to-T transitions (Fig. 1B), which is consistent with the mode of action of EMS (29), and these were widespread among the 145 mutants sequenced. Some small insertions and deletions (InDels) were also observed in coding regions, and these were more frequent in P. aeruginosa than in E. coli or K. pneumoniae (Fig. 1A).

10.1128/mSystems.00465-19.3 TABLE S2 E. coli mutants and their corresponding mutations. Download Table S2, XLSX file, 0.4 MB. Copyright © 2019 El Khoury et al. 2019 El Khoury et al. This content is distributed under the terms of the Creative Commons Attribution 4.0 International license.

10.1128/mSystems.00465-19.4 TABLE S3 K. pneumoniae mutants and their corresponding mutations. Download Table S3, XLSX file, 0.1 MB. Copyright © 2019 El Khoury et al. 2019 El Khoury et al. This content is distributed under the terms of the Creative Commons Attribution 4.0 International license.

10.1128/mSystems.00465-19.5 TABLE S4 P. aeruginosa mutants and their corresponding mutations. Download Table S4, XLSX file, 0.07 MB. Copyright © 2019 El Khoury et al. 2019 El Khoury et al. This content is distributed under the terms of the Creative Commons Attribution 4.0 International license.

**FIG 2:**
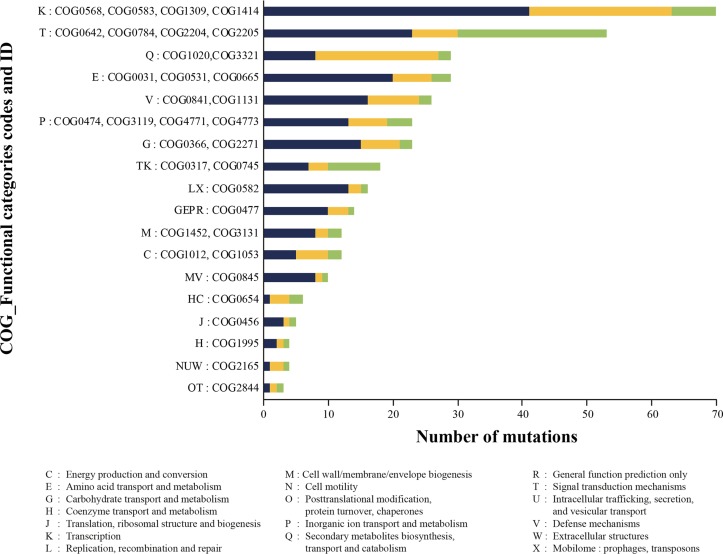
COG functional categories detected among the three species for the mutated genes. Mutated genes were classified into the appropriate COG, and the most common COGs shared by E. coli (blue), K. pneumoniae (orange), and P. aeruginosa (green) are shown.

**FIG 3:**
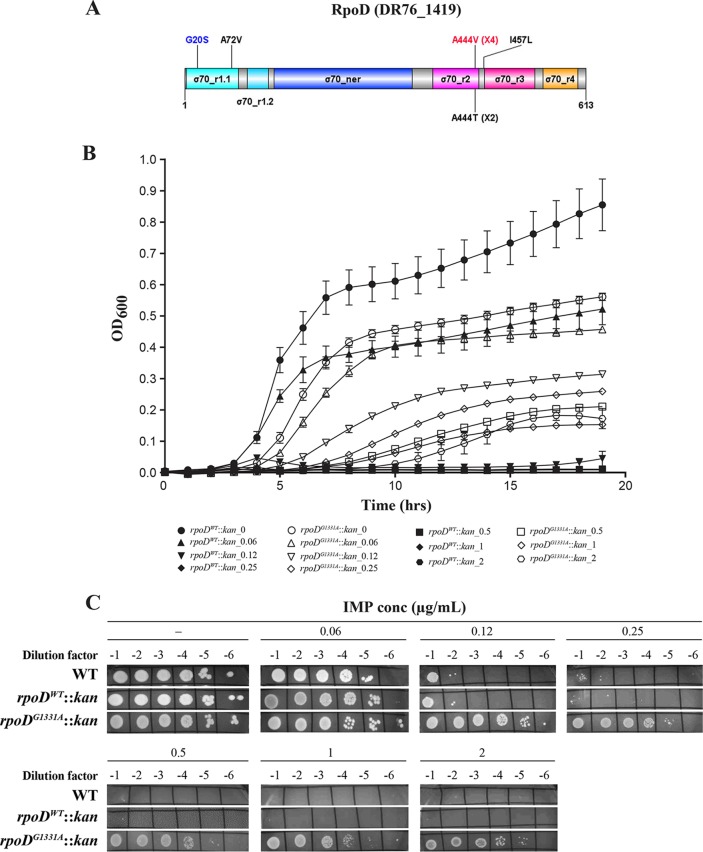
Validation of the role of RpoD in IMP susceptibility. (A) Schematic representation of the RpoD (DR76_1419) domains. The mutation marked in red was used to generate the single knock-in in E. coli ATCC 25922 (*rpoD*^G1331A^::*kan*). Numbers between parentheses indicate the recurrence of the mutation among clones. The G20S and E20K (not shown) substitutions were found in only one mutant of K. pneumoniae and one of P. aeruginosa, respectively. σ70_r, sigma 70_region; ner, nonessential region. (B) Growth curves of *rpoD*^WT^::*kan* and *rpoD*^G1331A^::*kan* in LB in the absence or presence of IMP at the indicated concentrations. Data shown represent averages ± standard errors of the means (SEM) of results from three biological replicates done in technical triplicate. (C) Cultures of E. coli ATCC 25922 WT, *rpoD*^WT^::*kan*, and *rpoD*^G1331A^::*kan* were serially diluted and spotted on LB agar plates in the absence (-) or presence of imipenem (IMP) at the indicated concentrations. Plates were incubated overnight at 37°C and photographed. Data shown are representative of results from three biological replicates.

We hypothesized that functional recurrence among mutated genes between species or clones would help in pinpointing the mutations that are the most relevant to IMP resistance. To ease comparisons among the three species, we relied classification using the Clusters of Orthologous Groups of proteins (COGs). The COG database is composed of over 4,600 specific functional COG descriptions grouped into 26 general category letter associations (30). We initially focused on the part of the COG descriptions corresponding to the mutated genes common to the three species, then on those shared by at least two species, and finally on the genes that are species specific but that were mutated in a higher number of clones. Thirty-five functional COG descriptions were found in common among the three species (Table S5). A third of these belonged to two general categories: (i) transcription mechanisms (K) and (ii) signal transduction mechanisms (T) (Fig. 2). The *rpoD* gene was the most prevalent in the COG category transcription, with mutations detected in a total of 10 mutants in the three species (Table S5). A C58T transition occurred in *rpoD* for one mutant each of K. pneumoniae and P. aeruginosa, leading to G20S and E20K amino acid substitutions, respectively, while eight E. coli mutants harbored mutations leading to an A72V, A444V, A444T, or I457L substitution (Fig. 3A). Additional genes coding for DNA-binding transcriptional regulators, sensor histidine kinases (HK), major facilitator superfamily transporters, and multidrug transporters were also mutated in the three species, but the mutations were often seen in a single mutant for each species and thus they were not further studied (Table S5).

10.1128/mSystems.00465-19.6 TABLE S5 Common COGs amongst the three species. Download Table S5, XLSX file, 0.03 MB. Copyright © 2019 El Khoury et al. 2019 El Khoury et al. This content is distributed under the terms of the Creative Commons Attribution 4.0 International license.

The E. coli and K. pneumoniae mutants shared 275 COG descriptions. Of these, 11 consisted of one-to-one matches of E. coli and K. pneumoniae proteins that shared at least 70% sequence identity (implying genuine functional similarity) and that were also mutated in at least two mutants in each species (Table 4). These 11 genes belonged to 7 general functional categories, the cell wall/membrane/envelope biogenesis category (M) being the most prevalent, with 4 mutated genes (Table 4). One of the 11 genes was *amiC* coding for an *N*-acetylmuramoyl-l-alanine amidase mutated in 9 E. coli and 26 K. pneumoniae mutants (Table 4). A majority of mutations localized to the AmiC domain of the protein, and several were nonsense mutations (see Fig. S1A in the supplemental material). Another gene was *nlpD*, mutated in 5 E. coli and 18 K. pneumoniae mutants (Table 4). This gene codes for the activator of AmiC (31). Similarly to *amiC*, several nonsense mutations were observed in *nlpD* (Fig. S1B). The third gene was *wecA*, coding for an undecaprenyl-phosphate alpha-*N*-acetylglucosaminyl 1-phosphate transferase mutated in 5 mutants of each species (Table 4). The last gene from the M category was *slt*, which codes for a soluble lytic murein transglycosylase. The *slt* gene was mutated in 5 mutants, a majority harboring nonsense mutations (Fig. S1C). The remaining 7 genes were not part of the cell wall biogenesis category and had unrelated functions (Table 4). These were shared by 4 to 9 E. coli and K. pneumoniae mutants (Table 4). For gene *clbJ* (COG1020, category Q), the E1684G mutation found in 15 mutants of K. pneumoniae was present also in the IMP-sensitive E. coli WT strain, making it less likely to contribute to IMP resistance.

**TABLE 4:** COG categories and functional descriptions of genes mutated in at least two mutants of E. coli and K. pneumoniae

COG category	COG description	Function	Gene ID^*a*^		Gene designation^*b*^
			E. coli^*b*^^,^^*c*^	K. pneumoniae^*b*^	
C	COG4656	Na^+^-translocating ferredoxin:NAD^+^ oxidoreductase Rnf	**DR76_3209** (2)	**DR88_4075** (2)	*rnfC*
G	COG2814	Predicted arabinose efflux permease	DR76_1590 (2)		*nanT*
			DR76_1762 (2)		
			DR76_4561 (2)		*araJ*
				DR88_690 (3)	

J	COG0445	tRNA U34 5-carboxymethylaminomethy modifying enzyme MnmG/GidA	***DR76_727*** (4)	**DR88_3339** (2)	*gidA*
	COG0215	Cysteinyl-tRNA synthetase	**DR76_4436** (2)	**DR88_4524** (3)	*cysS*
L	COG0847	DNA polymerase III	DR76_3797 (2)	DR88_1301 (2)	

M	COG0860	N-acetylmuramoyl-l-alanine amidase	***DR76_1787*** (9)	**DR88_2369** (26)	*amiC*
	COG1388	LysM repeat	***DR76_1882*** (5)	**DR88_2261** (18)	*nlpD*
	COG0472	UDP-N-acetylmuramyl pentapeptide phosphotransferase/ UDP-N-acetylglucosamine-1-phosphate transferase	***DR76_689*** (5)	**DR88_3479** (5)	*wecA*
	COG0741	Soluble lytic murein transglycosylase	***DR76_2503*** (3)	**DR88_109** (2)	*slt*
	COG0438	Glycosyltransferase involved in cell wall biosynthesis	DR76_4781 (3)		
			DR76_1541 (2)		
				DR88_1607 (3)	

NW	COG3188	Outer membrane usher protein FimD/PapC	DR76_1626 (2)		*papC*
			DR76_2428 (2)		*fimD*
			DR76_3348 (3)		*fimD*
			DR76_3932 (3)		*fimD*
				DR88_2135 (2)	*fimD*
				DR88_3815 (2)	
				DR88_397 (2)	*StfC*
P	COG4773	Outer membrane receptor for ferric coprogen and ferric-rhodotorulic acid	DR76_3822 (2)		*fhuE*
				DR88_4364 (2)	
Q	COG1020	Nonribosomal peptide synthetase component F	**DR76_2812** (2)	**DR88_1541** (15)	*clbJ*

R	COG2373	Uncharacterized conserved protein YfaS	**DR76_408** (2)	**DR88_2018** (2)	
	COG0612	Predicted Zn-dependent peptidase	DR76_3359 (3)	DR88_3068 (3)	

S	COG1649	Uncharacterized lipoprotein YddW	***DR76_3362*** (6)	**DR88_3813** (3)	
TK	COG0317	(p)ppGpp synthase/hydrolase	***DR76_839*** (2)	**DR88_3198** (2)	*spoT*
V	COG0841	Multidrug efflux pump subunit AcrB	DR76_4821 (7)		*MdtB*
			DR76_4822 (2)		*MdtC*
				DR88_665 (2)	
				DR88_584 (2)	*acrB*

a Gene accession numbers are indicated for E. coli ATCC 25922 and K. pneumoniae ATCC 13883. The number of mutants in which the genes were found mutated is indicated within parentheses.

b Gene designations in bold represent orthologues sharing at least 70% sequence identity at the protein level.

c Gene designations in italics represent genes that have been functionally tested for their role in resistance to IMP by individual transformation in E. coli ATCC 25922 WT (see Table 5).

10.1128/mSystems.00465-19.1 FIG S1 Schematic presentation of the domains of AmiC (A), NlpD (B), and Slt (C) and their respective mutations in E. coli (EC) and K. pneumoniae (KP). The Pfam protein family database was used to determine the domains of each protein, and DOG2.0 software was used for the visualization of the protein domain structures. Mutations marked in red are the mutations used to generate the single knock-in. Numbers between parentheses indicate the rate of recurrence of the mutations occurring two or more times. For Slt (C), the scheme represents the proteins in E. coli (EC) (121 Ins, W455*, and R475*) and K. pneumoniae (KP) (A152V and R376*). Download FIG S1, TIF file, 0.9 MB. Copyright © 2019 El Khoury et al. 2019 El Khoury et al. This content is distributed under the terms of the Creative Commons Attribution 4.0 International license.

P. aeruginosa mutants were fairly distinct compared to E. coli and K. pneumoniae mutants, and these were thus not directly compared to E. coli and K. pneumoniae mutants in 2-way comparisons.

### Species-specific mutations.

Recurrence among clones was previously shown to ease the identification of SNVs contributing to the resistance phenotype (26), so, to retrieve gene candidates specifically mutated in E. coli, we thus focused on those mutated in at least 3 clones (Table 1). The gene *yceG* encoding a cell division protein was the most prevalent among E. coli clones, with 15 SNVs (including 5 nonsense mutations) detected in 20 mutants (Table 1). The second most prevalent gene was DR76_2948 coding for a trehalose-6-phosphate synthase, with two mutations (D376N and P380L) found in a total of 14 E. coli mutants (Table 1). Seven different mutations (5 of which were nonsense) occurred in the *rne* gene for six mutants. This gene codes for RNase E. Four different mutations occurred in five mutants for gene DR76_475, encoding a glutamate racemase. Finally, the *tolA* gene, which is part of the Tol-Pal cell envelope complex, was mutated in three E. coli mutants, including mutants M24 and M45, which also hold a mutated *amiC* gene (Table 1). A more exhaustive list of mutations can be found in Table S6.

10.1128/mSystems.00465-19.7 TABLE S6 Common mutated genes amongst E. coli mutants. Download Table S6, XLSX file, 0.2 MB. Copyright © 2019 El Khoury et al. 2019 El Khoury et al. This content is distributed under the terms of the Creative Commons Attribution 4.0 International license.

The mutational landscape of P. aeruginosa was more limited than that determined for E. coli (Fig. 1A), so we lowered our cutoff for candidate genes to correspond to those mutated in at least two mutants (Table S7). All mutants had a mutation either in the porin OprD or in two-component systems (TCSs) consisting of a sensor HK and a response regulator (Table 2). For *oprD*, 20 different mutations were found in 28 P. aeruginosa clones, 16 of which led to a stop codon (Table 2). Every mutant with a nonsense mutation in *oprD* had an IMP MIC of 16 μg/ml, representing an 8-fold increase compared to WT P. aeruginosa (Table 2). This was also the case for mutant M34 harboring a Y343N substitution in OprD, while other *oprD* coding mutations (G183D, G402D, and S325F) were associated with lower IMP resistance levels (Table 2). For TCSs, 15 mutants had a mutation in the sensor HK A4W92_13070 (Table 2). This HK is part of a two-component signal transduction system for which A4W92_13065, mutated in 4 independent P. aeruginosa clones (Table 2), is the response regulator. Interestingly, mutants M6 and M9 had a R419H mutation in the HK A4W92_04840 (Table 2), the same change that occurred in mutants M1, M3, and M11 for the HK described above (A4W92_13070). Lastly, the sensor HK PhoQ (A4W92_05675) was mutated in P. aeruginosa clones M5 (Q258*) and M36 (E198K) (Table 2).

10.1128/mSystems.00465-19.8 TABLE S7 Common mutated genes amongst P. aeruginosa mutants. Download Table S7, XLSX file, 0.02 MB. Copyright © 2019 El Khoury et al. 2019 El Khoury et al. This content is distributed under the terms of the Creative Commons Attribution 4.0 International license.

### Phenotypic validation of mutations highlighted by Mut-Seq.

The role of specific mutations in resistance to IMP was tested in E. coli ATCC 25922 using a knock-in approach. This involved the transformation of WT cells with a DNA cassette made of a PCR fragment containing the mutation fused to a kanamycin (Kan) resistance gene flanked by FLP recombination target (FRT) sequences used for the removal of the selection marker by the FLP/FLPe recombinase. For the *rpoD* gene mutated in all species, we tested the G1331A transition that was detected in E. coli and that led to the A444V amino acid substitution (Table 5). This mutation conferred a 4-fold increase in IMP resistance in the *rpoD*^G1331A^::*kan* transformants compared to the *rpoD*^WT^::*kan* cells used as a control (we failed to remove the *kan* resistance marker in *rpoD*^G1331A^::*kan* despite several attempts) (Table 5). The transformant *rpoD*^WT^::*kan* had the same IMP MIC (0.25 μg/ml) as the E. coli ATCC 25922 WT. The A444V mutation allowed cells to sustain higher IMP concentrations than the control both in liquid medium (Fig. 3B) and on solid agar (Fig. 3C) but conferred a slight fitness cost in the absence of IMP (Fig. 3B). Interestingly, the *rpoD*^G1331A^::*kan* mutant had decreased susceptibility also for MEM (Table 5).

**TABLE 5:** Functional validation of mutation detected in *E. coli* IMP-resistant mutants

Gene ID^*a*^	Gene designation	Source^*b*^	Mutation		MIC (μg/ml)^*c*^	
			Nucleotide	Amino acid	IMP	MEM
Single knock-in						
ATCC 25922					0.25	0.03
DR76_475		M2	G761A	A254V	0.25	ND
**DR76_689**	*wecA*	M12	G118A	R40C	0.25–0.5	ND
**DR76_727**	*gidA*	M20	C1616T	A539V	0.25	ND
**DR76_839**	*spoT*	M14	G413A	A138V	0.25–0.5	ND
*DR76_1419* e	*rpoD*	M23	G1331A	A444V	1	0.06
**DR76_1787**	*amiC*	M20	G1204A	G402R	0.5	0.03
**DR76_1882**	*nlpD*	M48	C481T	Q161*	0.5	0.03
**DR76_2503**	*slt*	M14	C1423T	R475*	0.5	0.03
DR76_2948		M50	C1139T	P380L	0.25	ND
**DR76_3362**		M29	G1285A	A429T	0.25	ND
DR76_3827	*yceG*	M15	G274A	Q92*	0.25	ND
DR76_3839	*rne*	M3	C2323T	Q775*	0.5	0.03
DR76_4272	*tolA*	M11	C201T	M67I	0.5	0.03

Double knock-in						
Gene 1						
*amiC*				G402R		
Gene 2						
*slt*				R475*	1	0.06
*yceG*				Q92*	0.5	ND
*gidA*				A539V	0.5	ND

a Single knock-in, mutations in genes were transformed in an individual fashion; Double knock-in, mutations in two genes were transformed into the same E. coli ATCC 25922 cells. Gene IDs in bold had mutations in at least two E. coli and K. pneumoniae mutants (see Table 4). The gene in italics was mutated in E. coli, K. pneumoniae, and P. aeruginosa.

b The mutant whose genomic DNA was used to amplify the mutation by PCR to generate the knock-in cassettes.

c MICs were monitored with at least three biological replicates. For all differences of 2-fold or higher, there was no variability in the observed MICs. The *kan* resistance marker was removed from all transformants using the FLP/FLPe recombinase except for *rpoD*. ND, not determined.

Using the same knock-in approach, we also investigated the role in IMP resistance of mutations in genes shared by E. coli and K. pneumoniae mutants (Table 4). The G402R substitution in AmiC, a position mutated in two E. coli mutants and one mutant of K. pneumoniae (Fig. S1A), decreased the susceptibility to IMP by 2-fold when introduced into E. coli ATCC 25922 (Table 5). Similarly, the nonsense mutations Q161* and R475* in NlpD and Slt, respectively, increased the IMP MIC by 2-fold (Table 5). E. coli mutant M14 and K. pneumoniae mutant M17 had mutations in both *amiC* and *slt*, and the AmiC G402R and Slt R475* mutations were indeed additive in increasing the IMP MIC by 4-fold under conditions of cotransformation into E. coli ATCC 25922 (Table 5). This *amiC* and *slt* double knock-in also showed decreased susceptibility to MEM (Table 5). Despite being mutated in 5 mutants each of E. coli and K. pneumoniae (Table 4), the role of *wecA* mutations was less clear as the transformant for the R40C mutation (detected in E. coli mutant M12) was not conclusively altered for its IMP MIC (Table 5). Regarding the mutations in genes that are unrelated to cell wall biogenesis but that have at least 70% sequence identity between E. coli and K. pneumoniae (Table 4), we tested mutations A138V in SpoT, A429T in DR76_3362, and A539V in GidA, but, similarly to the results seen with *wecA*, none had a significant impact on the IMP MIC (Table 5).

We also tested some representatives of the most prevalent genes specifically detected in E. coli. The mutations in *yceG* and *rne* leading to the Q92* and Q775* nonsense mutations, respectively, as well as the P380L substitution in DR76_2948 and the M67I mutation in TolA, were independently transformed in E. coli ATCC 25922. The IMP MIC was increased by 2-fold (0.5 μg/ml) in the case of the *rne* and *tolA* knock-in (Table 5).

**FIG 4:**
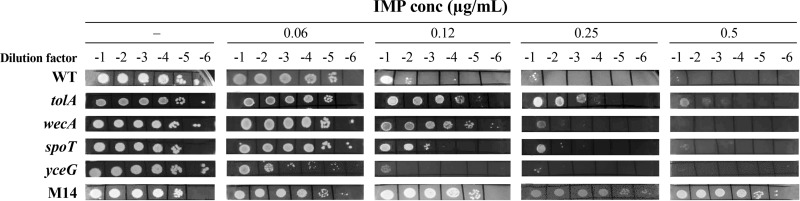
Validation of the roles of WecA and SpoT in IMP susceptibility in E. coli. Cultures of E. coli ATCC 25922 WT; of single knock-ins harboring TolA (M67I), WecA (R40C), SpoT (A138V), or YceG (Q92*) mutations; and of E. coli IMP-resistant mutant M14 were serially diluted and spotted on LB agar plates in the absence (-) or presence of imipenem (IMP) at the indicated concentrations. Plates were incubated overnight at 37°C and photographed.

The knock-ins for mutations in *wecA* and *spoT* described above had an ambiguous phenotype by MIC measurements, so we looked for subtler phenotypes by monitoring their growth by serial dilution on solid medium in the presence of IMP. As expected, the E. coli M14 mutant (harboring an A138V mutation in SpoT) grew until it reached the highest cell dilution and IMP concentration tested. In contrast, E. coli ATCC 25922 WT grew only to the 10^−1^ dilution on an agar plate supplemented with 0.25 μg/ml of IMP (its MIC) (Fig. 4). As a positive control for transformation, we used the M67I mutation in TolA. This mutation allowed cells to grow until they reached the 10^−2^ dilution at 0.5 μg/ml (Fig. 4), consistent with the MIC of this transformant (Table 5). Mutations R40C in WecA and A138V in SpoT had an intermediate phenotype in growing with more dilutions than the WT cells at 0.12 μg/ml IMP and at the 10^−1^ dilution at 0.25 μg/ml IMP (Fig. 4). Given its recurrence, we also tested the *yceG* gene but the Q92* mutation did not increase growth in the presence of IMP and was even detrimental under the conditions tested (Fig. 4).

## DISCUSSION

The use of whole-genome screens is now allowing holistic views of mechanisms of action and mechanisms of resistance against antimicrobial agents. We applied here a Mut-Seq screen (25, 26, 32) combining chemical mutagenesis and NGS to obtain clones of E. coli, K. pneumoniae, and P. aeruginosa with decreased susceptibility to IMP. The main advantage of Mut-Seq in comparison to other approaches such as step-by-step drug selection is the rapidity with which resistant mutants are obtained (24 h versus months), hence accelerating studies on the mode of action or mechanisms of resistance to antimicrobials. We posited that using diverse species and analyzing a large set of independent mutants would pinpoint shared pathways of resistance as well as species-specific traits. As expected from phylogeny, the response of E. coli to IMP shared more similarity with K. pneumoniae than with P. aeruginosa. Some responses were shared among the three species, and while we concentrated on the genes mutated in the greatest number of mutants, it is quite possible that mutant-specific genes are also important for IMP responses.

The two main categories of orthologous proteins shared among the three species were those corresponding to transcription and signal transduction mechanisms. The most prevalent gene common to the three species was *rpoD*, which codes for the σ^70^ factor that is associated with the core RNA polymerase complex for initiation of specific transcription (33). Mutations in *rpoD* were in distinct domains, and we validated experimentally the role of the A444V substitution detected in several independent E. coli mutants. This substitution occurred in a region of RpoD that is highly conserved, as it is part of the −10 promoter recognition helix binding (34). One current limitation of our work is the lack of validation of the role of *rpoD* directly in K. pneumoniae or P. aeruginosa. This type of effort could form the basis of further studies. While *rpoD* has never been shown to be involved with IMP resistance, sigma factors are well known to regulate a number of genes often associated with responses to stress (35). Further work may also provide insights into the downstream target gene(s) responsible for the decrease in IMP susceptibility. Many other genes (62 in E. coli and 39 in K. pneumoniae), including several that are strain specific, that are involved in transcription were mutated, and some may indeed help in the response to IMP. Among many of the TCSs mutated in each of the three species, *evgS* and *phoQ* were common (see Table S5 in the supplemental material). Knock-in of *evgS* or *phoQ* was not possible because of the gene arrangement and the close proximity of their respective regulators *evgA* and *phoP*.

IMP inhibits bacterial cell wall synthesis by binding to and inactivating the PBPs, with the highest affinity to PBP-1a, PBP-1b, and PBP-2 (36, 37), leading to rapid cell lysis and death (38). MEM also targets PBPs, with the highest affinity for *Pseudomonas* PBP-2 and PBP-3. While less active than IMP against enterococci, it is more active against P. aeruginosa (39). Our highly IMP-resistant P. aeruginosa mutants and our E. coli knock-ins with at least 4-fold resistance to IMP were all less susceptible to MEM (Table 3 and Table 5), demonstrating that our findings with IMP can be applied to MEM as long as the IMP MIC has reached a minimum threshold. This corroborates results of other studies demonstrating that IMP-resistant P. aeruginosa strains are usually cross-resistant to MEM as well (19, 20). Similarly, E. coli strains selected for MEM resistance showed decreased susceptibility to IMP (40). Consistent with IMP mode of action, genes from the category of cell wall and membrane biogenesis were among those most highly represented in E. coli and K. pneumoniae. Amidases such as AmiC split the peptidoglycan (PG) of daughter cells during cell division (37). These amidases are autoinhibited and AmiC is activated by NlpD, a lipoprotein anchored in the outer membrane (31). Mutations in both *amiC* and *nlpD* contributed to a decrease in susceptibility to IMP (Table 5). It was demonstrated that the Tol-Pal system is implicated in regulating cell wall cleavage during cell division by activating NlpD (41) as well as in the modulation of PG synthesis (42). TolA, part of the Tol-Pal complex, was found mutated in three mutants of E. coli, and the M67I mutation decreased the susceptibility of the WT strain by 2-fold (Table 5). Mutations in *amiC*, *nlpD*, and *tolA* are likely to be loss-of-function mutations, since *nlpD* and *amiC* have nonsense mutations or InDels in many mutants (see Fig. S1A and B in the supplemental material). Our observations are consistent with previous studies where mutants lacking *nlpD* or amidases and several lytic transglycosylases (see below) displayed a delayed lysis response to ampicillin (43, 44).

The PG is made of alternating *N*-acetylglucosamine (GlcNAc) and *N*-acetylmuramic acid (MurNAc) connected by a β-1,4-glycosidic bond (37). Lytic transglycosylases (LTs) cleave this glycosidic bond (37). E. coli has eight membrane-bound LTs (MLTs) and one soluble LT named Slt70 (37). Several of these LTs (MltA, MltB, MltD, and RlpA) were mutated in single E. coli or K. pneumoniae mutants and were not studied (Tables S2 and S3), but the *yceG* (MltG) gene was mutated in 20 E. coli mutants, including 5 with nonsense mutations (Table 1). The transformation of the mutation Q92* in the E. coli ATCC 25922 WT did not change the susceptibility of the strain to IMP, and it did not provide a growth advantage in the presence of IMP (Table 5) (Fig. 4). While we could not find a specific role of *yceG* in IMP resistance, its frequent mutation in E. coli suggests a role possibly in combination with other mutations. Indeed, MltG was shown to interact with PBP1b and in the absence of PBP1b, MltG was shown to be toxic (45). Since IMP inhibits PBP1b, it is possible that loss-of-function mutations in *yceG* are selected to limit its toxicity. Inhibition of PBPs by β-lactams leads to an accumulation of non-cross-linked PG, and Slt70 is the main enzyme responsible for destroying this nascent PG (46). Slt inactivation produced differential effects on β-lactam sensitivity depending on the genetic background (46–48). Here, we showed that a *slt* nonsense mutation (R475*) in E. coli ATCC 25922 decreased the susceptibility to IMP by 2-fold.

The response of P. aeruginosa to IMP differed extensively from the responses seen with the two *Enterobacteriaceae* species. The outer membrane permeability of P. aeruginosa is about 12-to-100-fold lower than that of E. coli (49), and this is probably due to a reduced number of general diffusion porins and the presence of a variety of specific porins such as OprD (16, 50). IMP penetrates the pseudomonal membrane through OprD (51). Resistance to IMP in P. aeruginosa can occur through loss of OprD, which has been reported to take place at the levels of transcription and translation (14, 52). Mutations resulting in a premature stop codon were found in a number of clinical isolates (53–56), several of which (W6*, Q19*, W65*, W138*, Q158*, W277*, Q295*, W339*, and W415*) were also detected in this study together with new ones (Q30*, Q67*, Q79*, and Q235*) (Table 2). Stop codons occurred at tryptophan or glutamine residues, representing the only two codons that can be changed to a stop codon through a single transition (along with one of the six codons for arginine) induced by EMS. TCSs are known to downregulate OprD and contribute to IMP resistance (57, 58). Among the P. aeruginosa ATCC 27853 mutants selected in this study, 19 mutants had a mutation either in the sensor HK gene (A4W92_13070) or in the gene encoding its response regulator (A4W92_13065) without having a mutated *oprD* gene (Table 2). Two independent mutants had a mutation in another sensor HK gene (A4W92_04840) (Table 2). These mutated HKs belong to the same clade as the ones known to regulate OprD (59). Two mutations (E198K and Q258*) in the sensor HK PhoQ (A4W92_05675) were detected in two independent clones (Table 2). A PhoQ-null mutant is resistant to polymyxin B and to aminoglycoside antibiotics (60–62), and PhoQ mutants were reported in P. aeruginosa clinical isolates resistant to polymyxin B (63) or colistin (polymyxin E) (64). Current understanding would suggest that the mutated HKs activate their respective response regulators and that they in turn downregulate *oprD*. Mutated HKs were also observed in E. coli and K. pneumoniae strains resistant to IMP (Table S5), highlighting the potential key role of HKs in IMP responses in Gram-negative bacteria.

Our chemogenomic screen performed with three bacterial species highlighted shared and species-specific responses to IMP. The most highly mutated genes encoded proteins involved in transcription, signal transduction, and membrane/cell envelope biogenesis. The number of mutants investigated allowed a holistic view of the response to IMP and enabled concentrating our functional work on the most frequently recurrent genes. Most mutations tested in E. coli were associated with a 2-fold difference in IMP susceptibility. This relatively low level of resistance may reflect more-subtle roles for the mutations, such as facilitating resistance emergence or compensating for fitness cost. Many other mutations are likely to be involved in response to IMP, and our data set can be useful to better understand IMP and to find strategies to restore carbapenem susceptibility in Gram-negative bacteria.

## MATERIALS AND METHODS

### Bacterial strains, plasmids, and growth conditions.

Bacterial strains and plasmids used in this study are listed in Table S8 in the supplemental material. Unless otherwise specified, E. coli (ATCC 25922), K. pneumoniae (ATCC 13883), and P. aeruginosa (ATCC 27853) were cultured on Luria-Bertani (LB) or nutrient agar (1.5%) and incubated at 37°C for 18 to 24 h. Liquid cultures were grown in LB for E. coli and in brain heart infusion (BHI) medium for K. pneumoniae and P. aeruginosa. Plasmid pRedET and the FRT-flanked PGK-gb2-kan cassette (catalogue number K006) and the enhanced FLP expression plasmid 707-FLPe (catalogue number A104) with a tetracycline resistance marker were obtained from Gene Bridges GmbH. IMP-monohydrate and MEM were purchased from Santa Cruz Biotechnology; all other chemicals were purchased from Sigma-Aldrich.

10.1128/mSystems.00465-19.9 TABLE S8 Strains and plasmids used in this study and primers used to generate the single knock-in and the double knock-in. Download Table S8, PDF file, 0.03 MB. Copyright © 2019 El Khoury et al. 2019 El Khoury et al. This content is distributed under the terms of the Creative Commons Attribution 4.0 International license.

### Antibacterial susceptibility testing.

Analyses of the IMP or MEM MICs for the WT strain of each of the three species and for their respective mutants and optimization of the EMS concentrations (Table S8) were performed by microdilution in 96-well plates according to recommendations from the CLSI. The IMP or MEM MICs for the single or double knock-ins were determined by macrodilution. All MICs were determined from at least three independent biological replicates, each replicate being further assessed in technical duplicates.

### Chemical mutagenesis and selection of antibiotic-resistant mutants.

Optimization was performed by testing different concentrations of EMS equivalent to 4×, 8×, or 16× its MIC for each of the three species for either 10 or 20 min. We selected conditions that allowed treated cells to reach an optical density at 600 nm (OD_600_) of 0.5 in less than 6 h. For K. pneumoniae and P. aeruginosa, this represented an EMS concentration equivalent to 4 times their MIC (0.024 g/ml and 0.012 g/ml, respectively) maintained for 10 min. For E. coli, we used an EMS concentration equivalent to 8 times its MIC (0.048 g/ml) maintained for 20 min. The minimum concentration of IMP used for selection was determined as the concentration at which growth occurred in the presence of IMP for the mutagenized populations but not for the nonmutagenized control populations. This represented IMP concentrations equivalent to 16× and 20× the MIC in the case of E. coli (4 and 5 μg/ml) and between 2× and 4× the MIC for both K. pneumoniae (2 and 4 μg/ml) and P. aeruginosa (4 and 8 μg/ml). No clones survived beyond these concentrations. The detailed protocol was as follows: the overnight (ON) cultures of the strains were diluted and incubated at 37°C with shaking (220 rpm) until they reached an OD_600_ of 0.5. Each culture was separated into two tubes of 10 ml. We added EMS to one of the tubes at the appropriate concentration. Cultures were incubated for 10 min (K. pneumoniae and P. aeruginosa) or 20 min (E. coli) at 37°C. Cultures were then diluted by half using ice-cold medium and then further diluted by 1/10 before being incubated until an OD_600_ of 0.5 was reached. The cultures were centrifuged at 4,000 rpm for 5 min, and then the pellet was resuspended in 200 μl of the culture medium. Ten-fold dilutions (from 10^−3^ to 10^−7^) were spread on agar plates to allow colony counting.

In order to select mutants resistant to IMP, 100-μl volumes of the mutagenized cultures were spread on agar plates containing an increasing concentration of the antibiotic (between 2× and 20× the MIC depending on the species). Agar plates were incubated overnight at 37°C, and colonies were counted the next day. This protocol was performed for each strain in duplicate.

### Extraction and quantification of DNA.

Genomic DNA (gDNA) was extracted using a Wizard Genomic DNA purification kit (Promega) according to the manufacturer’s protocol. The purity of the gDNA was analyzed using a NanoDrop spectrophotometer. Quantification was performed by fluorescence detection using a QuantiFluor One double-stranded DNA (dsDNA) system (Promega).

### DNA sequencing.

Libraries were produced from 0.8 ng of gDNA using a Nextera XT DNA Library Prep kit (Illumina) according to the manufacturer’s protocol. Libraries were verified by the use of model 2100 Bioanalyzer high-sensitivity DNA chips (Agilent) and quantified by the use of a QuantiFluor One dsDNA system (Promega). Libraries were sequenced on an Illumina HiSeq 2500 system (101-nt paired-end sequencing) at a final concentration of 8 pM.

### Mut-Seq data analysis.

Sequence reads were aligned to the E. coli ATCC 25922 (BioProject accession no. PRJNA244551), K. pneumoniae ATCC 13883 (PRJNA244567), and P. aeruginosa ATCC 27853 (PRJNA316664) genomes using bwa-mem software (65). The seed length was 32, and 2 mismatches were allowed within the seed; the maximum number of mismatches allowed was 4. Read duplicates were marked using Picard (http://broadinstitute.github.io/picard/), and calling of SNVs and InDels was done using GATK software (66). Mutations in common with the WT strain sequence examined in parallel were excluded, and the remaining mutations (i.e., mutant-specific mutations) were annotated. To ease the comparisons among the three species, the common COGs (30) were determined using the workflow of COGsoft (https://sourceforge.net/projects/cogtriangles/files/).

### Generation of knock-ins in E. coli.

We first transformed E. coli ATCC 25922 with the expression plasmid pRedET as recommended by the manufacturer (Gene Bridges GmbH). Mutated PCR fragments of the genes of interest were amplified from the appropriate E. coli mutant and fused to the FRT-flanked PGK-gb2-kan cassette before being transformed in the E. coli strain containing the pRedEt plasmid. This strategy was previously described by Sukhija et al. and Pyne et al. (67, 68). l-Arabinose was added to induce the expression of the Red/ET recombination proteins, and transformants were selected with Kan (40 μg/ml). Colonies were analyzed by colony PCR and sequencing. All primers used to generate the single knock-ins and to check the right integration of the gene replacement cassette are listed in Table S8. The enhanced FLP conditional expression plasmid 707-FLPe was transformed into the strain containing the gene replacement cassette so that recombination would occur at the FRT sites. PCR was used to confirm both the removal of the Kan cassette by FLP recombination and the presence of the knock-in mutation.

### Testing the effect of mutations on the growth of E. coli.

Cultures (OD_600_ = 0.9) were serially diluted (10^−1^ to 10^−6^) in LB, and 5-μl volumes were spotted on freshly prepared LB agar plates supplemented with different concentrations of IMP. Plates were incubated at 37°C overnight, and they were photographed the next day using an AlphaImager system (Alpha Innotech).

For determination of the growth curve in liquid medium, 5 μl of 1 × 10^7^ CFU/ml was inoculated in 200 μl of LB medium in the absence or presence of IMP at concentrations of 0.03 to 4 μg/ml in 96-well plates. The plate was incubated at 37°C, and the OD_600_ was read each 30 min after shaking for 10 s using a Cytation 5 multimode reader. Each assay was done in technical triplicate and biological triplicate.

### Data availability.

The WGS data have been deposited in the SRA database, and the accession numbers are listed in Table S9.

10.1128/mSystems.00465-19.10 TABLE S9 Accession numbers of E. coli, K. pneumoniae, and P. aeruginosa mutants in the SRA database. Download Table S9, XLSX file, 0.02 MB. Copyright © 2019 El Khoury et al. 2019 El Khoury et al. This content is distributed under the terms of the Creative Commons Attribution 4.0 International license.
