# Supplementary material for: Chemogenomic Screen for Imipenem Resistance in Gram-Negative Bacteria
Source: mSystems. 2019 Nov 19;4(6):e00465-19. doi: 10.1128/mSystems.00465-19 (PMC6867876; doi:10.1128/mSystems.00465-19)
Supplement: TABLE S1 [file mSystems.00465-19-st001.pdf]

Table S1. MICs of IMP for *K. pneumoniae* ATCC 13883 mutants and amino acid substitutions detected in genes shared with *E. coli* mutants.

Strains[a]	Gene ID	DR88_2369	DR88_2261	DR88_3479	DR88_3813	DR88_109	DR88_3339	DR88_3198
	Gene Symbol	*amiC*	*nlpD*	*wecA*	-	*slt*	*gidA*	*spoT*
	MIC (μg/mL)[b]	Amino acid subtitutions[c]						
WT	1							
M1	2	P194L						
M2	2							
M3	2		Q90*					
M4	2	G205E						
M5	2			W129*				
M7	2	P194L						
M8	2	R238C						
M10	2	R249C						
M11	2		Q154*					
M12	2		Q140*					
M13	2	Q119*						
M14	2	P368L						
M15	2	E373K						
M16	2	T328I						
M17	2	Q254*				R376*		
M18	2		W259*					
M19	2		W259*					
M20	2		W129*					
M21	2	G402E						
M22	2		W129*		P22S			D667N
M24	2		R363H					
M25	2	G336E		H282				
M27	2		Q80*					
M29	2	G198S			W346*			
M30	2	Q392*						
M31	2	G197S						
M32	2		R363H					
M33	2		Q154*				L297F	
M34	2		Q67*					
M35	2		Q174*					
M36	2	W21*						
M37	2	Q254*						
M38	2	Q325*						
M39	2	G336E						
M40	2		R363C					
M41	2	G18D						
M42	2		Q65*					
M43	2	S263L						
M44	2		W129*					
M45	2	R238C						
M6	4	D149N	W259*					
M9	4	P365S		G52D				
M23	4	G195R		A302T				
M26	4		S368F	P48L	V366M	A152V		
M28	4	Q169*					R256C	P298L

[a] Mutants are listed in ascending order of MIC of IMP.

[b] MICs were monitored with at least three biological replicates.

[c] Mutations correspond to amino acid substitutions and numbers refer to amino acid position in the protein. Asterisks denote stop codons.
