# Supplementary figures and images for: Chemogenomic Screen for Imipenem Resistance in Gram-Negative Bacteria
Source: mSystems. 2019 Nov 19;4(6):e00465-19. doi: 10.1128/mSystems.00465-19 (PMC6867876; doi:10.1128/mSystems.00465-19)

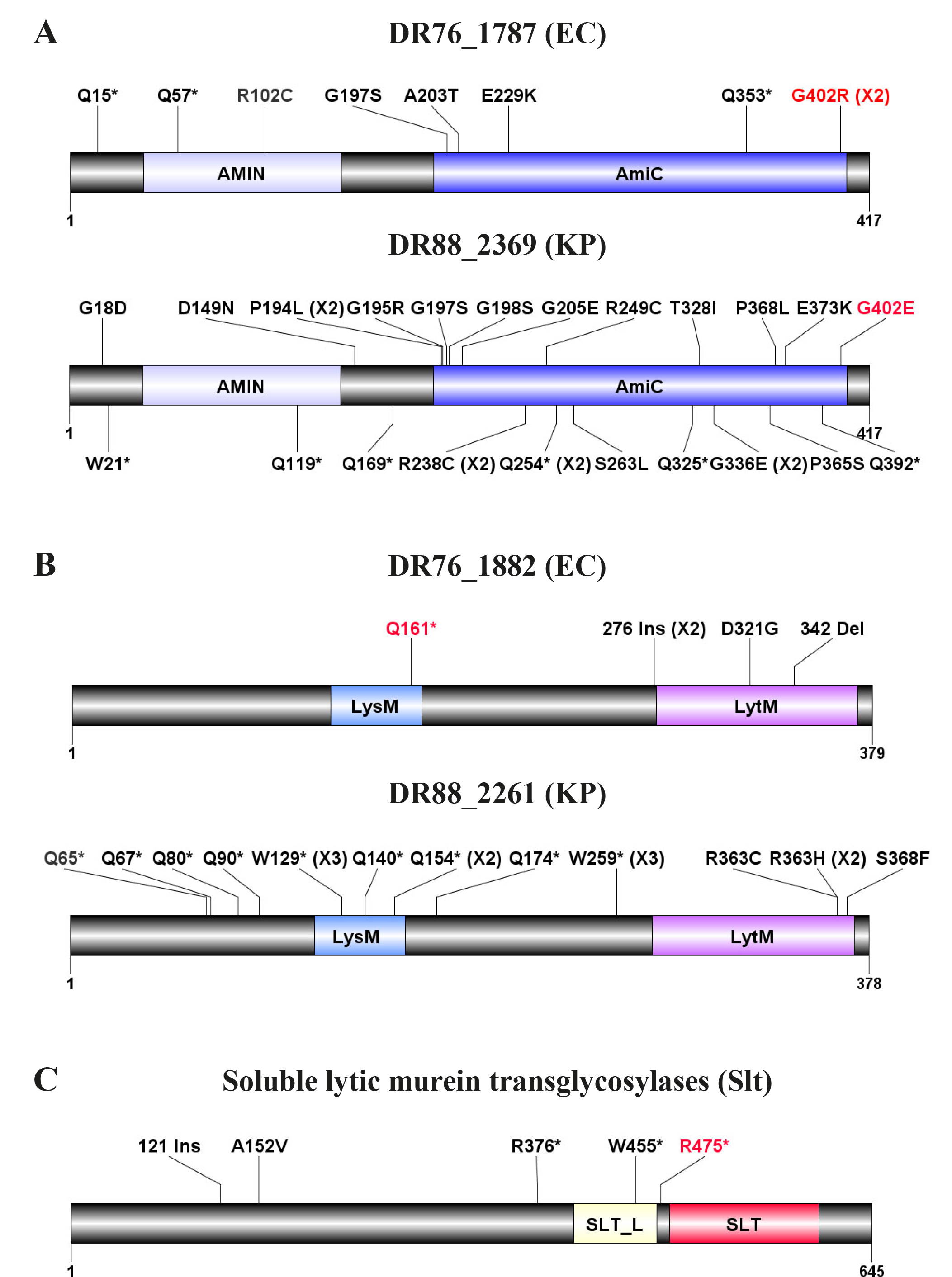

Supplement: FIG S1 [file mSystems.00465-19-sf001.tif]
