# Supplementary material for: Chemogenomic Screen for Imipenem Resistance in Gram-Negative Bacteria
Source: mSystems. 2019 Nov 19;4(6):e00465-19. doi: 10.1128/mSystems.00465-19 (PMC6867876; doi:10.1128/mSystems.00465-19)
Supplement: TABLE S8 [file mSystems.00465-19-st008.pdf]

Table S8. Strains and plasmids used in this study and primers used to generate the single and double knock-ins

Strain number	Species	MIC of IMP (μg/mL) [a]	MIC of EMS (g/mL) [a]	Source
ATCC 13883	*K. pneumoniae*	1	0.006	Kindly provided by Dr Michel G. Bergeron
ATCC 27853	*P. aeruginosa*	2	0.003	
ATCC 25922	*E. coli*	0.25	0.006	ATCC

Plasmids	Resistance marker	
pRedET	Tetracycline	Gene Bridges GmbH
707-Flpe		
pGem ®-T Easy	Ampicillin	Promega

Gene ID	Gene Symbol	Mutant_Source of the mutated PCR fragment	Nucleotide substitution	Primers	5'-3'
**FRT-scar down-stream of the gene**					
DR76_475	NA	M2	G761A	Frd_DR76_475	ATGGCTACCAAACTGCAGGACGGGAA
				Rv_DR76_475_Kan	***CGCCCTTTAGTGAGGGTTAATT***TCAATCTAAAACTGCCAGTTTTTCGAGC
				Frd_DR76_475_Kan	GCTCGAAAAACTGGCAGTTTTAGATTGA***AATTAACCCTCACTAAAGGGCG***
				Rv_Up_475_Kan	TTTCTAACCGCGCAACATTCAACCAAA***TAATACGACTCACTATAGGGCTC***
				Frd_DR76_475_seq	CGCCGTTGTTACGCCGTGCT
				Rv_DR76_475_seq	CCGTATCTTCGAGTGCCCACACA
DR76_727	*gidA*	M20	C1616T	Frd_DR76_727	CGAGGCAATCACCATGTTTTATCCGG
				Rv_DR76_727_kan	***CGCCCTTTAGTGAGGGTTAATT***TTATGCGCTACGACGCAGCATACCCTGT
				Frd_DR76_727_Kan	ACAGGGTATGCTGCGTCGTAGCGCATAA***AATTAACCCTCACTAAAGGGCG***
				Rv_Up_727_Kan	GGGTGCATACCAGGCATTTTTAATGCG***TAATACGACTCACTATAGGGCTC***
				Frd_DR76_727_seq	CCCAGGTCTTTCTCAAGCCGACA
				Rv_DR76_727_seq	AGGAGAGTTTGTTGAGCACGGTGA
DR76_1787	*amiC*	M20	G1204A	Frd_DR76_1787	CAGCTTAGATTTCTTTTGCTCGGGAGAGGC
				Rv_DR76_1787_Kan	***CGCCCTTTAGTGAGGGTTAATT***TTATCCCCTTCTCGCCAGCGTCGCCCCATC
				Frd_DR76_1787_Kan	GATGGGGCGACGCTGGCGAGAAGGGGATAA***AATTAACCCTCACTAAAGGGCG***
				Rv_Up_1787_Kan	AAATATAAATTCATTGAATAAATAAAAACA***TAATACGACTCACTATAGGGCTC***
				Frd_DR76_1787_seq	TCCCTCGACCAGCTCGGTTT
				Rv_ DR76_1787_seq	TGCGTATACATTACACGGAATTGTGCT
DR76_1882	*nlpD*	M48	C481T	Frd_DR76_1882	GGTTATTAACCAATTTTTCCTGGGGGATAA
				Rv_DR76_1882_Kan	***CGCCCTTTAGTGAGGGTTAATT***TTATCGCTGCGGCAAATAACGCAGCGGGTT
				Frd_DR76_1882_Kan	AACCCGCTGCGTTATTTGCCGCAGCGATAA***AATTAACCCTCACTAAAGGGCG***
				Rv_Up_1882_Kan	ACATTCAAGCAAAAGCCTGGTTCCGCCGAT***TAATACGACTCACTATAGGGCTC***
				Frd_DR76_1882_seq	TGACGCAGCTGGACGAAGGC
				Rv_DR76_1882_seq	GCCTTTTCGTCAAAAACCTCAACTCCG
DR76_2503	*slt*	M14	C1423T	Frd_DR76_2503	ACCCTAATACGGTACTGACCTTCGCGC
				Rv_DR76_2503_Kan	***CGCCCTTTAGTGAGGGTTAATT***TCAGTAACGACGTCCCCATTCCGTGGCG
				Frd_DR76_2503_Kan	CGCCACGGAATGGGGACGTCGTTACTGA***AATTAACCCTCACTAAAGGGCG***
				Rv_Up_2503_Kan	TACGATAGCATATCATAAACGTGCGGA***TAATACGACTCACTATAGGGCTC***
				Frd_DR76_2503_seq	CGGCGGAACAACTTTGCCGA
				Rv_DR76_2503_seq	CGGTAAATGGAGATCGTTTTGGTAGGC

Continued next page

Table S8. *Continued*

Gene ID	*Gene Symbol*	Mutant_Source of the mutated PCR fragment	Nucleotide substitution	Primers	5'-3'
**FRT-scar down-stream of the gene**					
DR76_2948	NA	M50	C1139T	Frd_DR76_2948	CAGGAGTGATGACTATGAGTCGTTTAGTCGTAG
				Rv_DR76_2498_Kan	***CGCCCTTTAGTGAGGGTTAATT***CTACGCAAGCTTAGGAAAGGTAGCAACT
				Frd_DR76_2498_Kan	AGTTGCTACCTTTCCTAAGCTTGCGTAG***AATTAACCCTCACTAAAGGGCG***
				Rv_Up_2498_Kan	GTTCCACTTACGGGAGATTAACCGCAC***TAATACGACTCACTATAGGGCTC***
				Frd_DR76_2948_seq	CCGGCTGGGCGGAATGTCAG
				Rv_ DR76_2948_seq	AGCTGCGCCGATGCTTGAAGA
DR76_3362	NA	M29	G1285A	DR76_3362_Frd	ATTCATCGCTCCCTTTTTCG
				Rv_DR76_3362_Kan	***CGCCCTTTAGTGAGGGTTAATT***TTAACTGCCCCAACGACTTTGCAGATAG
				Frd_DR76_3362_Kan	CTATCTGCAAAGTCGTTGGGGCAGTTAA***AATTAACCCTCACTAAAGGGCG***
				Rv_Up_3362_Kan	GGCTTGTTACAAAGTAAGAATGGGAGT***TAATACGACTCACTATAGGGCTC***
				Frd_DR76_3362_seq	TGCCCTGCCCTTTATTCTCTATGCT
				Rv_DR76_3362_seq	GCGACTTAGGACAGTTTCAATCTACGC
DR76_3839	*rne*	M3	C2323T	Frd_DR76_3839	ACACCAGCCGCTCCTGCCCAACCTGGG
				Rv_DR76_3839_Kan	***CGCCCTTTAGTGAGGGTTAATT***TTACTCAACAGGTTGCGGACGCGCAGGA
				Frd_DR76_3839_Kan	TCCTGCGCGTCCGCAACCTGTTGAGTAA***AATTAACCCTCACTAAAGGGCG***
				Rv_Up_3839_Kan	CCAGGGCTTGATTGCTTGAGCTAATTA***TAATACGACTCACTATAGGGCTC***
				Frd_DR76_3839_seq	CCCAGTCTGTTTCTCTGATAATTGCGCT
				Rv_DR76_3839_seq	GCTCGCTTTAAAACATATCATGAAACTGGG
**FRT-scar up-stream of the gene**					
DR76_689	*wecA*	M12	G118A	Frd_Kan_dn_DR76_689	TGCTAATAATTTTCTCTGAGAGCATGCATT***AATTAACCCTCACTAAAGGGCG***
				Rv_Kan_DR76_689	ATCAGTACTCACTGTCAGTAAATTCAC***TAATACGACTCACTATAGGGCTC***
				Frd_Kan_DR76_689	***GAGCCCTATAGTGAGTCGTATTA***GTGAATTTACTGACAGTGAGTACTGAT
				Rv_DR76_689	TTATTTGGTTAAATTGGGGCTGCCACC
				Frd_DR76_689_seq	TGCTGCGCTGGCGATGTTAGG
				Rv_DR76_689_seq	TCGAGCTCCACTCCTGGCGA
DR76_839	*spoT*	M14	G413A	Frd_Kan_dn_DR76_839	GGTCGTCGTTAATCACAAAGCGGGTCGCCC***AATTAACCCTCACTAAAGGGCG***
				Rv_Kan_DR76_839	CTGATTCAGGCTTTCAAACAGATACAA***TAATACGACTCACTATAGGGCTC***
				Frd_Kan_DR76_839	***GAGCCCTATAGTGAGTCGTATTA***TTGTATCTGTTTGAAAGCCTGAATCAG
				Rv_DR76_839	CACGCGGCATTCGGGCGAGCA
				Frd_DR76_839_seq	AGGCGGAAAGGATCCGCTGG
				Rv_DR76_839_seq	GTACGCCAACGGCATCTGCG
DR76_3827	*yceG*	M15	G274A	Frd_Kan_dn_DR76_3827	CCACTTTGTGAGCGCCCGAATTAGTC***AATTAACCCTCACTAAAGGGCG***
				Rv_Kan_DR76_3827	CAAGATTATCAATAACACTTTTTTCAT***TAATACGACTCACTATAGGGCTC***
				Frd_Kan_DR76_3827	***GAGCCCTATAGTGAGTCGTATTA***ATGAAAAAAGTGTTATTGATAATCTTG
				Rv_DR76_3827	TTACTGCGCATTTTTTTCCTTAAGCACTTTCAG
				Frd_DR76_3827_seq	ACGGAATGCTGTGCGGCTAAT
				Rv_DR76_3827_seq	TCGGCAAGTTGCGTACCGCC
DR76_4272	*tolA*	M11	C201T	Frd_Kan_dn_DR76_4272	TAACAGGCGAACAGTTTTTGGAAACCGAGA***AATTAACCCTCACTAAAGGGCG***
				Rv_Kan_DR76_4272	GTCGTTTTGTTCGGTTGCCTTTGACAC***TAATACGACTCACTATAGGGCTC***
				Frd_Kan_DR76_4272	***GAGCCCTATAGTGAGTCGTATTA***GTGTCAAAGGCAACCGAACAAAACGAC
				Rv_DR76_4272	TTACGGTTTGAAGTCCAATGGCGCG
				Frd_DR76_4272_seq	CACCAGAGCAGGTGGTGGCG
				Rv_DR76_4272_seq	GGCCCCGCCCACTGGAAAG

Continued next page

Table S8. *Continued*

**FRT-flanked PGK-gb2-kan cassette down-stream of the gene**					
DR76_1419	*rpoD*	M23	G1331A	Frd_DR76_1419	TAAGTGTGGATACCGTCTTATGGAGCAAAA
				Rv_DR76_1419_kan	***CGCCCTTTAGTGAGGGTTAATT***TTAATCGTCCAGGAAGCTACGCAGCACT
				Frd_DR76_1419_Kan	AGTGCTGCGTAGCTTCCTGGACGATTAA***AATTAACCCTCACTAAAGGGCG***
				Rv_Up_1419_Kan	GGCGTAACACCTGATCCGGCCTACCGA***TAATACGACTCACTATAGGGCTC***
				Frd_DR76_1419_seq	GCACTGAGAGGCAGCGGCAA
				Rv_DR76_1419_seq	AGGCGTTGGCGATTCTGGGC

[a] MICs were monitored with at least three biological replicates
